# Digital mental health for postpartum women: perils, pitfalls, and promise

**DOI:** 10.1038/s41746-023-00756-4

**Published:** 2023-01-26

**Authors:** Natalie Feldman, Sarah Perret

**Affiliations:** 1 Department of Psychiatry, Brigham and Women’s Hospital, 75 Francis Street, Boston, MA 02115 USA; 2 Harvard Medical School, 25 Shattuck Street, Boston, MA 02115 USA; 3 Department of Medicine, Brigham and Women’s Hospital, 75 Francis Street, Boston, MA 02115 USA; 4 Division of Digital Psychiatry, Beth Israel Deaconess Medical Center, Boston, MA 02115 USA

**Keywords:** Health care, Health services

## Abstract

Postpartum mental health conditions are a public health concern, affecting a large number of reproductive-age women and their families. Postpartum depression alone affects at least 14% of new mothers and their families. However, very little has been written about how advances in digital mental health can benefit women in the postpartum period, or how those advances may poorly serve this vulnerable population. This manuscript takes a high-level view of the advances in different areas of digital mental health, including telehealth, apps, and digital phenotyping. In this comment, we explore ways in which digital interventions for postpartum mental health may help with connection to treatment, accessibility, agency, and ease of access. We also note particular concerns for how digital postpartum mental health may encounter issues of low-quality resources, ethical considerations, and equity considerations. We provide suggestions for how to leverage the promise and avoid the pitfalls of digital mental health for postpartum women.

## Introduction

Postpartum mental health conditions are a public health concern, affecting many reproductive-age women and their families. The Centers for Disease Control and Prevention (CDC) estimated that symptoms of postpartum depression, the most commonly studied postpartum mental heath condition, were found in about 13% of women with a recent live birth^1^. Globally, that number may be even higher for postpartum depression, and is less well-defined for other postpartum mental health conditions^2^. Extensive research has shown the negative impacts of untreated postpartum mental health conditions on both mother and child^3^. Women with postpartum depression have been found to have worse physical health, more difficulties in social relationships, and a higher risk of engaging in substance use^3^. Maternal depression has also been associated with childhood developmental changes; although the results here are less clear, studies suggest there may be impacts in areas including emotional development, physical growth, and fine motor and language development^4–6^. Postpartum depression is also linked with an increase in mortality: suicide accounts for 1 in 5 deaths in postpartum women^7^.

The COVID-19 pandemic has exacerbated the need for mental health treatment^8^. In particular, the pandemic has increased the acute need for care for women in the postpartum period. A recent meta-analysis found higher rates of depression and significantly higher rates of anxiety in perinatal women, including postpartum women, compared to pre-pandemic levels^9^. The expansion of telehealth during the pandemic has resulted in increased reliance on technology for mental health care^10^. However, these changes have not been evenly distributed. While more people overall have used digital mental health services, populations who are disproportionately affected by social determinants of health may actually have had less access to their doctors during this time^11^. Additionally, despite the increased need for mental health care, the World Health Organization (WHO) found that 75% of mental health, substance use, and neurologic services suffered partial or complete disruption in 2020 and 2021^12^. Given the acute need and current lack of accessibility, novel solutions are needed to provide mental health care to women in the postpartum period. Digital mental health technologies, including digital phenotyping, telehealth, mental health apps, and others, have the potential to fill this gap between the need for treatment and access to care. However, there are particular risks to using novel digital mental health technologies in this population.

## Promise

One area of promise for digital tools in postpartum mental health is closing the gap between screening and treatment. While significant attention has been paid to the importance of screening for postpartum depression^13^, a systematic review of English language studies found that on average only 22% of women who screen positive for postpartum depression are connected to care^14^. According to the Substance Abuse and Mental Health Services Administration (SAMHSA) workforce report from 2020, over 4 million more behavioral health practitioners are needed^15^, which is likely fueling the gap between screening and care. Due to the lack of connection to care and shortage of providers, innovative solutions are desperately needed to expand treatment to more women. At their best, mental health apps can help deliver evidence-based interventions to women who are on a waiting list for a provider, augment traditional care so that fewer visits are needed, or even provide peer support or psychoeducation in place of traditional care.

Digital mental health solutions may also help with accessibility for new mothers. Telehealth has received positive attention during the pandemic^16^ for expanding accessibility for postpartum women, who may find that telehealth relieves barriers such as finding childcare or not wanting to take young children into a medical facility. The flexibility of apps to supplement care could make treatment more accessible than telehealth alone, particularly since 85% of women own a smartphone^17^. For busy new parents, the ability to log into an app at 4 AM while breastfeeding, rather than trying to shift a tenuous nap schedule to accommodate a doctor’s appointment, may be a godsend. Additionally, nearly half of women experience traumatic childbirth^18^, and returning to a doctor’s office at the site of that trauma may be upsetting; remote care could allow them to process that experience in their own time, rather than either feeling triggered or avoiding medically necessary care. Other developing modalities may also give postpartum women agency over their mental health, a critical piece of trauma-informed care^19^.

By examining some existing evidence-based interventions for postpartum mental health, we can see how these different modalities could allow new mothers and their teams to create a system of care that best works for their needs and availability. By combining traditional appointments, synchronous support interventions such as 7Cups, a peer support platform which has been studied in postpartum women^20^, and asynchronous support like the MumMoodBooster internet CBT program^21^, we may be able to build a treatment plan that fits each new mom, rather than asking patients to conform to a preset model of care.

Finally, digital phenotyping may offer a novel way to make it even easier for postpartum women to access mental health care. Digital phenotyping can use passive data (such as movement data or language content) to screen for mental health conditions without someone having to actively seek help. It also has the potential to provide precision medicine for the field of psychiatry^22^, better predicting what treatments will work for a particular patient. Although research in digital phenotyping in postpartum women is limited, one study successfully used digitally administered measures to generate three phenotypes of postpartum women, which allowed for early detection of postpartum depression^23^. A review of digital phenotyping for mood disorders in a general population found that digital phenotyping not only has the potential for early detection of mood disorders, but also the capability to capture the nuances of distinct phenotypes of mental illness within a large class. This may in turn improve predictions of treatment response and outcomes^24^. Within the postpartum population, digital phenotyping could give postpartum individuals tools to monitor and assess their mental health. An exciting possibility of digital phenotyping is that it may change the problematic dynamic wherein new mothers in mental health crisis are expected to find the wherewithal to reach out and find help. With digital phenotyping, people in crisis could be identified and offered help, which they could accept or refuse.

As a hypothetical example, a digital phenotyping program could use passive data collected via smartphone or smart watch, such as daily movement or text patterns, to identify women whose movement or texting patterns suggested high risk of depression or mania. This program could then provide these women with information about postpartum depression or mania, which might encourage postpartum women in crisis to seek help sooner. Additionally, rather than waiting a week or more between appointments to receive self-report data on how a patient is feeling that day, digital phenotyping could collect daily passive data across that entire week and display a graph to help both doctor and patient assess whether a given treatment is working. Digital phenotyping could also be used to predict, based on a new patient’s passive data patterns, whether a given patient would respond better to sertraline or citalopram.

## Pitfalls

While postpartum digital mental health is rife with promise, there are also significant pitfalls to note. The first is that not all digital resources are good resources. In a different manuscript, one of the authors of this piece found that searching commercial app stores for “postpartum depression” generated hundreds of results. Most of those apps were not relevant to postpartum mental health, and almost none of those that were relevant were backed by research. Even apps which explicitly targeted postpartum mental health were rarely able to cite any evidence either for their recommendations or for the apps themselves^25^. While a doctor in a traditional clinic waits for FDA approval before prescribing a new medication for postpartum depression, new apps in this space can be available to consumers with little to no content oversight. Increasingly, resources like the American Psychiatric Association’s App Guide^26^ strive to help doctors recommend evidence-based apps. However, as new apps in this space continue to be rapidly developed^25^, providers may struggle to become proficient at navigating these resources, and postpartum women may be left to fend for themselves. We must remember that just because an intervention is convenient, or technologically innovative, doesn’t mean it works.

It’s also important to consider the ethical ramifications of digital interventions for postpartum women. Digital phenotyping may show promise, but it also raises questions about women’s autonomy and how new moms are viewed. Digital phenotyping of new moms can be seen as implying that postpartum women are unstable, can’t be trusted to know what they need, and need to be surveilled. In light of the history of women feeling unheard, unconsidered, or disenfranchised by medicine, this surveillance may read as dystopian^27,28^. It’s also critical to acknowledge that this could feel particularly menacing for women of color, as research has shown that child-welfare services are more likely to become involved with parents of color^29^. This could in turn discourage women from admitting that they’re struggling. The question of autonomy over one’s health information also brings in issues of privacy, such as the period-tracking app Flo, which settled allegations of illegally sharing users’ personal health information^30^. To navigate the history of health disparities in women, particularly women of color^31^, issues of autonomy in postpartum digital phenotyping will need to be handled with significant care.

Innovators in postpartum digital mental health must also remain vigilant against the possibility that the benefits of these advances are not well distributed. Social determinants of health (SDOH) have been extensively linked with disparities in mental health care outcomes^32^. In one example, a recent study highlighted numerous ways in which individual and structural racism limited Black women’s access to reproductive health services, including via financial and insurance limitations, having access to lower quality health-care services, and feeling dismissed or invalidated by providers^33^. Digital mental health may offer some promise in lowering these barriers for postpartum women; for example, women without access to affordable childcare or transportation could theoretically have greater access to care from home. However, the experience of telehealth in the pandemic suggests that expanding access and addressing barriers is more difficult than simply instituting digital interventions: as discussed above, populations with a high risk of health-care disparities may actually have less access to their doctors in the era of telehealth^11^. If digital mental health interventions are not offered equally to postpartum women of color, are not affordable or adequately covered by insurance, or feature providers or attitudes which are invalidating towards women of color, they risk becoming a niche benefit available only to women of a certain race or class rather than a way of improving postpartum mental health across the board.

## Harnessing promise and avoiding pitfalls

There are several steps that can help maximize the promise and avoid the pitfalls of digital postpartum mental health. Reproductive-age women, the primary stakeholders, should be involved in all stages of the development process. This can help combat the history of women’s disenfranchisement in medicine^34^. Stakeholders will be even more key in order to increase access to care in underserved postpartum populations. Postpartum women from underserved populations should be invited to weigh in from the earliest conception phases of new digital mental health interventions. This will help ensure that these innovations reach a greater proportion of postpartum women who need them, and that interventions aimed at increasing access will actually target the barriers that these women face. This may include solutions for access to technology, resources for digital literacy, or other changes that help historically disenfranchised women feel safe using these tools. The Mothers and Babies Internet Course is a great example of how to do this well; the authors built an internet-based PPD prevention intervention based on evidence-based modalities, and then surveyed a diverse group of mothers about how the intervention could be improved to better reflect the needs of women from diverse backgrounds^35^.

Additionally, effectively expanding postpartum mental health treatment to digital platforms calls for leveraging the strengths of academia and technology together. The LAMP platform, which is currently being piloted in a postpartum population, is an excellent model for how this can work well. LAMP is an open-source platform that can be customized for different conditions, allowing innovators to move fast without reinventing the wheel. It has academic backing from the LAMP consortium. It is set up to utilize passive digital phenotyping data as well as active surveys and cognition tests, all of which can be used to augment traditional postpartum psychiatric care^36^. If we leverage the speed of industry, the rigor of academia, and the expertise of postpartum women themselves, digital postpartum mental health can live up to its bright promise.
